# The use of microfluidic spinning fiber as an ophthalmology suture showing the good anastomotic strength control

**DOI:** 10.1038/s41598-017-16462-7

**Published:** 2017-11-24

**Authors:** DoYeun Park, In Sung Yong, Kyong Jin Cho, Jie Cheng, Youngmee Jung, Soo Hyun Kim, Sang-Hoon Lee

**Affiliations:** 1 KU-KIST Graduate School of Converging Science and Technology, Korea University, 145 Anam-ro, Seongbuk-gu, Seoul 02841 Republic of Korea; 2 Department of Bio and Brain Engineering, KAIST, Daejeon, 34141 Republic of Korea; 3 Department of Ophthalmology, College of Medicine, Dankook University, 119 Danaeo-ro, Dongnam-gu, Cheonan-si, Chungcheongnam-do 31116 Republic of Korea; 4 MOE Key Laboratory of Spectrochemical Analysis & Instrumentation, Collaborative Innovation Center of Chemistry for Energy Materials, Key Laboratory for Chemical Biology of Fujian Province, State Key Laboratory of Physical Chemistry of Solid Surfaces, College of Chemistry and Chemical Engineering, Xiamen University, Xiamen, 361005 China; 5 Biomaterials Research Center, Korea Institute of Science and Technology, 5, Hwarang-ro 14-gil, Seongbuk-gu, Seoul 02792 Republic of Korea; 6 School of Biomedical Engineering, College of Health Science, Korea University, 145, Anam-ro, Seongbuk-gu, Seoul 02841 Republic of Korea

## Abstract

Adjusting the mechanical strength of a biomaterial to suit its intended application is very important for realizing beneficial outcomes. Microfluidic spinning fiber have been attracting attention recently due to their various advantages, but their mechanical strength has unfortunately not been a subject of concentrated research, and this lack of research has severely limited their applications. In the current work, we showed the mechanical properties of microfibers can be tuned easily and provided a mathematical explanation for how the microfluidic spinning method intrinsically controls the mechanical properties of a microfluidic spinning fiber. But we were also able to adjust the mechanical properties of such fibers in various other ways, including by using biomolecules to coat the fiber or mixing the biomolecules with the primary component of the fiber and by using a customized twisting machine to change the number of single microfiber strands forming the fiber. We used the bundle fiber as an ophthalmology suture that resulted in a porcine eye with a smoother post-operative surface than did a nylon suture. The results showed the possibility that the proposed method can solve current problems of the microfibers in practical applications, and can thus extend the range of applications of these microfibers.

## Introduction

A poor match between the mechanical properties of a biomaterial and those of a body part (*e.g*. cells, tissue, and organs) into or onto which the biomaterial is implanted or applied, could cause a transfer of undue stress to the body part, and hence a distortion of its structure and degradation of its function^1–3^. For example, nylon or mersilene, each of which is commonly used as a suture material in keratoplasty and is much stronger than the cornea, could cause post-keratoplasty astigmatism due to the resulting mismatch between a suture and the cornea. This mismatch may lead to a structural distortion in the cornea, specifically the formation of an oval opening, which causes astigmatism^4,5^. In this regard, the mechanical properties of a biomaterial should be designable according to the mechanical properties of the body part with which it is meant to interact. (Table 1, refs^6–16^).

**Table 1:** Mechanical properties of human tissues.

Tissues	Modulus	Tensile Stress	Strain at Break	Reference
**Soft tissues**	[MPa]^a^	[MPa]	[%]	
Smooth muscle, relaxed	0.006	—^b^	300	^5^
Skeletal muscle, relaxed	0.012	—	300	^5^
Pericardium	20.4/1.9^c^	—	34.9/1.1	^6^
Aorta	3.13	0.3~0.8	50–100	^7, 8^
Skin	10	1~20	30~70	^8, 9^
Patellar Tendon (29–50 years old)	660/266	64.7/15	14/6	^10^
Anterior cruciate ligament (21–30 years old)	345/22.4	36.4/2.5	15/0.8	^11^
Cornea	0.1579 (50–64 years old)	3.81	—	^12^
Brain	0.009/0.0008*^e^	—	—	^13^
**Hard tissues**	[GPa]^d^	[MPa]	[%]	
Cortical Bone	17~24	90~130	1~3	^14^
Cancellous Bone	0.1~4.5	10~20	5~7	^14^
Cartilage	0.001~0.01	10~40	15~20	^14^
Femur (20–39 years old)	17.6	124/1.1	1.41	^15^

^a^Megapascal; ^b^a value not provided in the reference; ^c^(average)/(standard deviation); ^d^Gigapascal; ^e^Simulated value.

Among various fiber-form biomaterials^17–21^, microfluidic spinning microfibers have been widely used in biomedical applications such as tissue engineering^22–25^ and wound dressing^26^ due to their extraordinary features such as their ability to guide cell growth^27–29^, their large surface-to-volume ratios^30–32^, and the various ways their surfaces can be modified. Among the diverse approaches for fabricating microfibers, microfluidics has recently attracted much attention because of its advantages in providing simple, rapid, and spatiotemporal control over the composition of the material along the microfiber, and the ability of this method to encapsulate a cell in the microfiber^22,24,33,34^. However, the importance of the mechanical properties of resulting fibers exposed to physiological conditions has been overlooked. Considering that a substantial part of our body consists of fibrous materials and that their mechanical properties vary, biomaterials such as the above-mentioned sutures need to be produced with various well-controlled mechanical properties. That is, an ability to choose the values of the mechanical properties of microfluidic spinning microfibers would widen the range of their applications.

**Figure 1:**
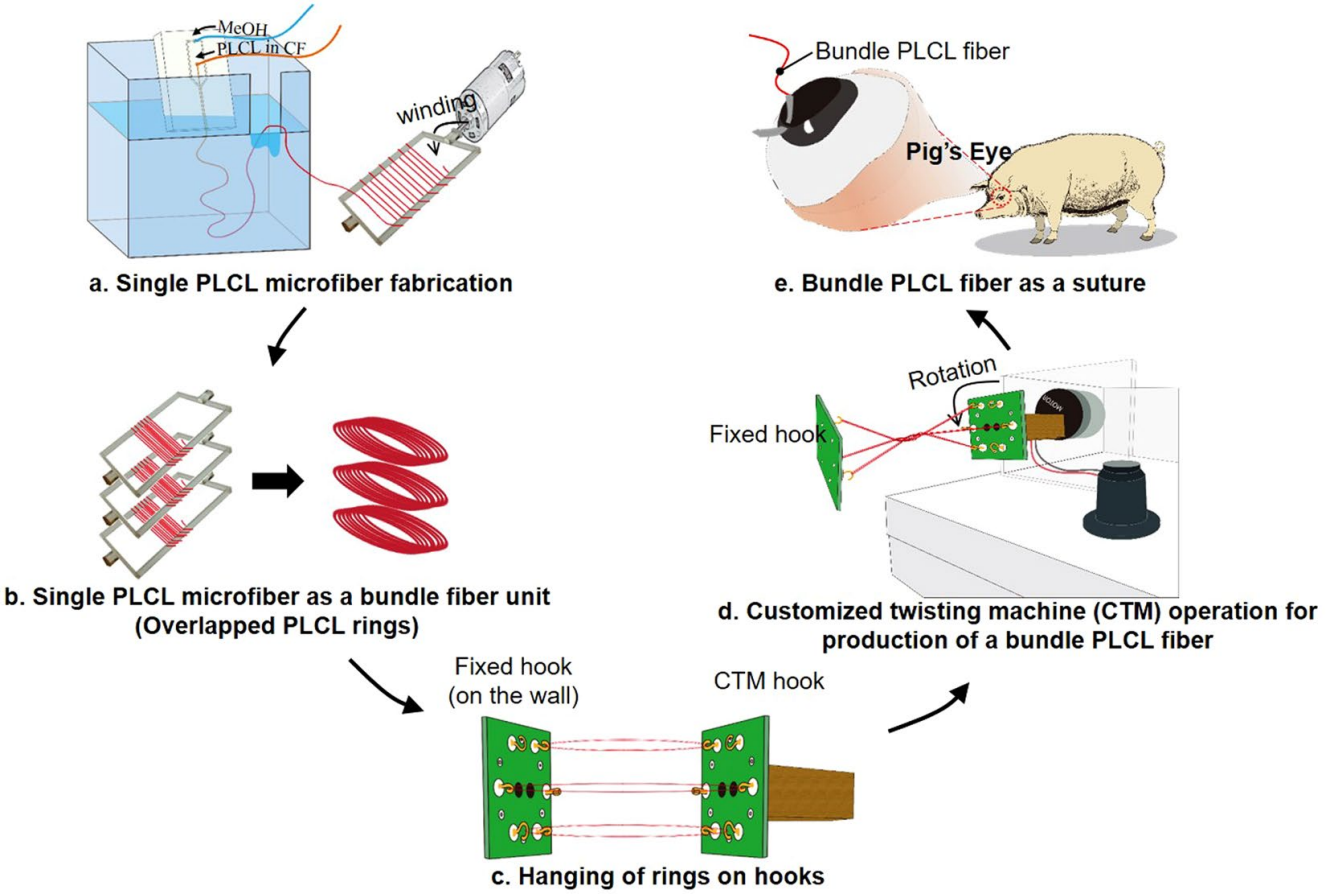
Overview of the experimental process. (**a**) Collection of a single PLCL microfiber spun using a microfluidic chip to make (**b**) overlapped PLCL rings. (**c**) The overlapped PLCL rings were then hung on a fixed hook and customized twisting machine (CTM) hook and stretched to be flattened. (**d**) Operation of the CTM caused the production of a bundle PLCL fiber. (**e**) We used the bundle PLCL fiber as an ophthalmology suture to stitch up an incision on a porcine eye.

Here, we suggested a novel system to control the mechanical properties of fiber-form biomaterials for better compatibility on the anastomosis. As shown in Fig. 1, we first showed that the conventional microfiber spinning chip has an intrinsic ability to control the mechanical properties of single microfibers, and provided a mathematical explanation for this ability (Fig. 1a). A customized twisting machine (CTM) was built to form bundle fibers based on single microfibers. The measurements of the mechanical properties of the bundle fiber were assessed according to the number of single fibers contained in the fiber (Fig. 1b–d). In addition, the effects of mixing a biomolecule with a single microfiber, or coating the biomolecule on the bundle fiber, on the mechanical properties of the bundle fiber were determined. Finally, we stitched up an incision made in a porcine cornea using a poly(l-lactic-co-ε-caprolactone) (PLCL) bundle fiber to check the effects of the mechanical properties of the suture on the tissue (Fig. 1e). These experiments were carried out with the pig eye itself, not with eyes of pigs alive. Optical coherence tomography (OCT) images of the operated-upon eyes were acquired to observe the effect of matching the mechanical properties of the biomaterials with those of the target tissue, and we found that good anastomotic strength control by suggested method would lead us to better post-operative results. This fabrication method has broadened the applications of microfluidic spinning microfibers, especially to their use as surgical sutures.

## Materials and Methods

### Mechanical property control of single PLCL microfiber

#### Fabrication of single PLCL microfiber using microfluidic chip

In order to demonstrate the ability of a microfiber-spinning chip imparting various mechanical properties onto the microfibers, we first fabricated the microfiber using microfluidic spinning chip as done before^22–24,33–36^. We introduced a PLCL solution (in chloroform) as a sample flow and methanol as a sheath flow into a core inlet and sheath inlet, respectively, to form a laminar flow. Then, solvents of each solution (chloroform for the PLCL solution, and methanol) diffused into each other, which caused the precipitation of PLCL since PLCL is insoluble in methanol. As shown in Fig. 1a, we set the direction of the fiber extrusion to be parallel to the direction of gravity, by hanging the chip in the bath, in order to prevent the interference of fiber fabrication due to the sinking of chloroform within the channel. To minimize the change in fabrication conditions caused by the hydrostatic force of methanol, we positioned the drainage of the bath to be 1 mm higher than the position of the end of the outlet channel. We only used microfibers extruded from the outlet in the shape of a straight line and that appeared white when observed by the naked eye, together denoted as a ‘feasible condition’, for subsequent experiments. In the non-feasible conditions, microfibers extruded from the outlet were transparent. Here, the conditions tested were the molar ratio of PLLA to that of PLCL (5:5 and 7:3), and the core flow rate when the sheath flow rate was 20 mL/h, 30 mL/h, and 40 mL/h.

#### Equation explaining the mechanical property control of single PLCL microfiber

To explain these phenomena, we suggested a simple equation for the relationship between length density (ρ_*l*_) of PLCL molecules in the microfiber and the initial Young’s modulus (iYm). iYm was determined from the slope of the Stress-Strain (SS) curve between 0% and 20% of the strain. The equation is

1 $${\rho }_{l}\propto \frac{{Q}_{PLCL,k}\times {A}_{k}}{{Q}_{MeOH,k}}$$

where k indicates a specific condition, Q_PLCL_ is the core flow rate, Q_MeOH_ is the sheath flow rate, and A_k_ is the cross-sectional area of the microfiber. The derivation of this equation is described as followed:

Based on our hypothesis that the distribution of PLCL molecules in the axial direction of the microfiber affects the iYm of the microfiber, we compared length density with iYm. The derivation of the equation (1) for the length density of the microfiber resulted from the following steps.

1. Length density of the microfiber is
  2 $${{\rm{\rho }}}_{{\rm{l}}}=\frac{{\rm{M}}}{{\rm{l}}}$$
  where l is the length of the microfiber that yields the same mass in each condition, and _M_ is the mass of the microfiber.
2. In our experimental conditions, the number of PLCL molecules is proportional to mass and the volume of the microfiber, so equation (2) can be modified to be
  3 $${{\rm{\rho }}}_{{\rm{l}}}=\frac{{\rm{M}}}{{\rm{l}}}\propto \frac{{{\rm{V}}}_{{\rm{constant}}}}{{\rm{l}}}=\frac{{{\rm{Q}}}_{{\rm{PLCL}},k}\times {{\rm{t}}}_{{\rm{k}}}}{{\rm{l}}}$$
  where *k* denotes a specific experimental condition, and t_k_ is the spinning time for making a fiber of the same mass (V_constant_) in condition *k*.
3. l can be expressed as a function of t_k_ and the speed at which the fiber is extruded from the outlet in condition k (v_throughput, k_), which could be expressed as
  4 $${\rm{l}}={{\rm{t}}}_{{\rm{k}}}\times {{\rm{v}}}_{{\rm{throughput}},{\rm{k}}}$$
4. In the case of microfluidic fiber spinning, since the sample flow rate is much less than the sheath flow rate, equation (4) can be modified to be
  5 $${\rm{l}}={{\rm{t}}}_{{\rm{k}}}\times \frac{{{\rm{Q}}}_{{\rm{MeOH}},{\rm{k}}}}{{{\rm{A}}}_{{\rm{k}}}},\,({{\rm{v}}}_{{\rm{throughput}},{\rm{k}}}\approx \frac{{{\rm{Q}}}_{{\rm{MeOH}},{\rm{k}}}}{{{\rm{A}}}_{{\rm{k}}}})$$
  where A_k_ is the cross-sectional area of the microfiber in condition k.
5. Substituting equation (5) into equation (3) yields

6 $${\rho }_{{\rm{l}}}\propto \frac{{{\rm{Q}}}_{{\rm{P}}{\rm{L}}{\rm{C}}{\rm{L}},{\rm{k}}}\times {{\rm{t}}}_{{\rm{k}}}}{{{\rm{t}}}_{{\rm{k}}}\times \frac{{{\rm{Q}}}_{{\rm{M}}{\rm{e}}{\rm{O}}{\rm{H}},{\rm{k}}}}{{{\rm{A}}}_{{\rm{k}}}}}=\frac{{{\rm{Q}}}_{{\rm{P}}{\rm{L}}{\rm{C}}{\rm{L}},{\rm{k}}}\times {{\rm{A}}}_{{\rm{k}}}}{{{\rm{Q}}}_{{\rm{M}}{\rm{e}}{\rm{O}}{\rm{H}},{\rm{k}}}}$$

That is, the length density of a PLCL molecule of a microfluidically spun microfiber is proportional to the ratio of sample flow rate to sheath flow rate and is proportional to A_k_.

Experimentally determined iYm values and length densities calculated according to above equation were plotted for microfibers made using core flow rates of 6, 7, 8, 9, and 10 μL/min with sheath flow rates of 20 mL/h, 30 mL/h, and 40 mL/h for the (5:5)PLCL microfibers. (We investigated that the feasible condition of core flow rate of 6, 7, 8, 9, and 10 μL/min for sheath flow rate of 20 mL/h was also a feasible condition for sheath flow rate of 30 mL/h and 40 mL/h).

### Mechanical property control of bundle PLCL fiber

#### Fabrication of bundle PLCL microfiber using the CTM

Bundle fibers were constructed from single microfibers whose mechanical properties differed from one another using the CTM. Immediately after single microfibers were extruded from the microfiber-spinning chip, we collected them around an acryl spool (20 mm width) to form a ring of overlapping PLCL microfibers (Fig. 1b). The length of the outlet channel and flow condition were set appropriately (i.e., 9 mm) for imperfect precipitation of chloroform to occur within the chip, and the remaining chloroform on the surface of the microfiber was simply collected and allowed to bond together. Then, rings of overlapping PLCL microfibers were loaded onto a CTM. The CTM was designed to have three parts: a controller used to determine the twisting direction and speed, hooks used to load various numbers of microfibers, and a motor to drive the twisting. The bundle fibers were made by loading a specified number of single microfiber strands; in this regard, we also made a fixed hook on the wall, which was used to maintain the distance from the CTM hook to fixed hook in order to maintain the twisting condition. One side of each overlapped ring was hung on a hook of the CTM, and another side of the ring was hung on a fixed hook, far enough away (20 mm) from the CTM hook to flatten the ring (Fig. 1c). The twisting rate, time and direction were set on the controller as 60 rpm, two minutes, and a clockwise, respectively. Then, the operation of the motor formed the bundle PLCL fiber (Fig. 1d). Since it was the ring of overlapping microfibers that was loaded, the number of microfiber strands contained in the bundle fiber was double the number of times the single microfiber was wound around the spool. Therefore, since we wound a single microfiber around the acryl spool either 4, 8, or 12 times, the twisted bundle fibers wound up having had eight (4 × 2), sixteen (8 × 2), or twenty-four (12 × 2) microfiber-strands (‘twisted group’). We also loaded either two or three twisted bundle fibers onto the CTM, so the final bundle fibers in the former case contained either sixteen (2 × 4 × 2), thirty-two (2 × 8 × 2), or forty-eight (2 × 12 × 2) single microfiber strands (‘wound group’).

#### Coating the bundle fiber with an alginate solution and mixing hdECM with PLCL

PLCL fibers were coated with alginate by passing the bundle fiber soaked in a 2% CaCl_2_ solution (in methanol) through a drop of a 2% alginate solution three times (Fig. S2). The method used to mix PLCL with hdECM was developed by Tae Hee Kim of Korea University (data not published yet). Details of this method are not shown here but will be shown in another paper. However, briefly, porcine heart (obtained from Myung-in bio, Republic of Korea) and 100% acetic acid was prepared for mixing. We mixed the hdECM solution with the PLCL solution in chloroform to obtain an hdECM-to-PLCL mass ratio of 1:9 or 2:8. It took about a day using a stirring bar to achieve mixed state before being used.

### Morphology observation using SEM

SEM images of each fiber fabricated under feasible and non-feasible conditions were acquired to observe their morphologies and measure their diameters. All fibers were placed in a methanol bath overnight after the fabrication process and, to remove all evaporative elements, were placed in a vacuum for at least 30 minutes before being placed in the SEM apparatus.

### Characterization of the mechanical properties of a single PLCL microfiber and bundle fiber

We quantified the mechanical properties of single microfibers and bundle fibers by analyzing SS curves. To obtain SS curves, we loaded fibers onto an Instron 5966 machine. But first, in order to load the fibers, we made a nick on a wooden rod and put the sample fiber in the wooden rod. Then, the wooden rod with fibers was loaded onto Instron tensile zig, which is the part to fix the sample in the tensile test, to avoid having the fibers slip and hence avoid the formation of distortions in the SS curve (Fig. S3). Single microfibers and bundle fibers loaded onto the Instron machine were set as a geometry of circular shape and irregular shape, respectively, and pulled at a rate of 10 mm/min until they broke. Another pulling rate of 5 mm/min was also applied to twisted groups to see the effect on the mechanical response. Since it was relatively difficult to say the bundle fibers are perfect circular shape, we set them as irregular shape and the cross-sectional area was determined by first measuring the diameters of its single component microfibers, then calculating the individual cross-sectional areas of the individual microfibers from these diameters, and finally adding up the values of these individual cross-sectional areas. All experiments including fabrication and mechanical properties measurement were conducted under constant temperature (20 °C) and relative humidity (40%). Then, the SS curve for each condition was obtained. The elongation at break, ultimate tensile strength (UTSH) were measured, and ultimate tensile stress (UTSS) and iYm were calculated. The UTSS was determined from the UTSH divided by cross-sectional area of the fiber while the iYm was determined from the slope of the SS curve between 0% and 20% of the strain. Each condition was tested with six independent samples. Analyzing SS curve was performed using Origin 8.0 program.

### DSC analysis

Thermal properties of both pure PLCL microfibers and PLCL microfibers containing hdECM were measured to verify the role of hdECM in improving the mechanical properties of PLCL microfibers. Measurements were taken using a Q10 DSC instrument (TA Instruments, New Castle, DE, USA) with nitrogen as a purge gas. Microfiber samples each with a mass of about 5–10 mg were prepared. The results were obtained from conditions including a heating rate of 20 °C/min and temperature range of −20 °C to 150 °C.

### *Ex vivo* surgery

In order to verify the advantages of being able to determine the mechanical properties of PLCL fibers, *ex vivo* ophthalmology surgery experiments were performed on pig eyes itself, not with eyes of pigs alive. Excised pig eyes were obtained from a local slaughterhouse. They were washed clearly and transported in phosphate buffer saline at a temperature of 5 °C. Based on the conditions of the system developed for controlling the mechanical properties of biomaterials, we performed cornea surgery using a fabricated bundle fiber whose mechanical properties relatively well matched the mechanical properties of the porcine cornea (Fig. 1e). The Young’s modulus and UTSS of the porcine cornea were reported to be 0.1449 MPa^9^ and 3.70 MPa^10^, respectively. We used 8 times wound group bundle fiber that included hdECM in its microfibers and that showed an initial Young’s modulus of 0.125 MPa and UTSS of 50 MPa. While by no means could this fiber, according to these values, be considered a perfect match for the porcine cornea, they were more mechanically favorable for the cornea than is a nylon suture^37–39^. We then connected the bundle PLCL fiber to the surgical needle, detached from the surgical suture product (Ailee Co., Ltd, lot number 0300177), to use it as a surgical suture. An incision was made on the cornea and *ex vivo* surgery performed with the bundle PLCL suture and a nylon 10–0 suture (Ethicon, ethilon10–0) as a control. We injected saline through the incision site to verify that no leakage happened after surgery. One hour after surgery, we used Cirrus^TM^ HD-OCT (ZEUS) of the cornea to observe the effect of the suture on the anastomosis and, in turn, the morphology of the cornea.

## Results

**Figure 2:**
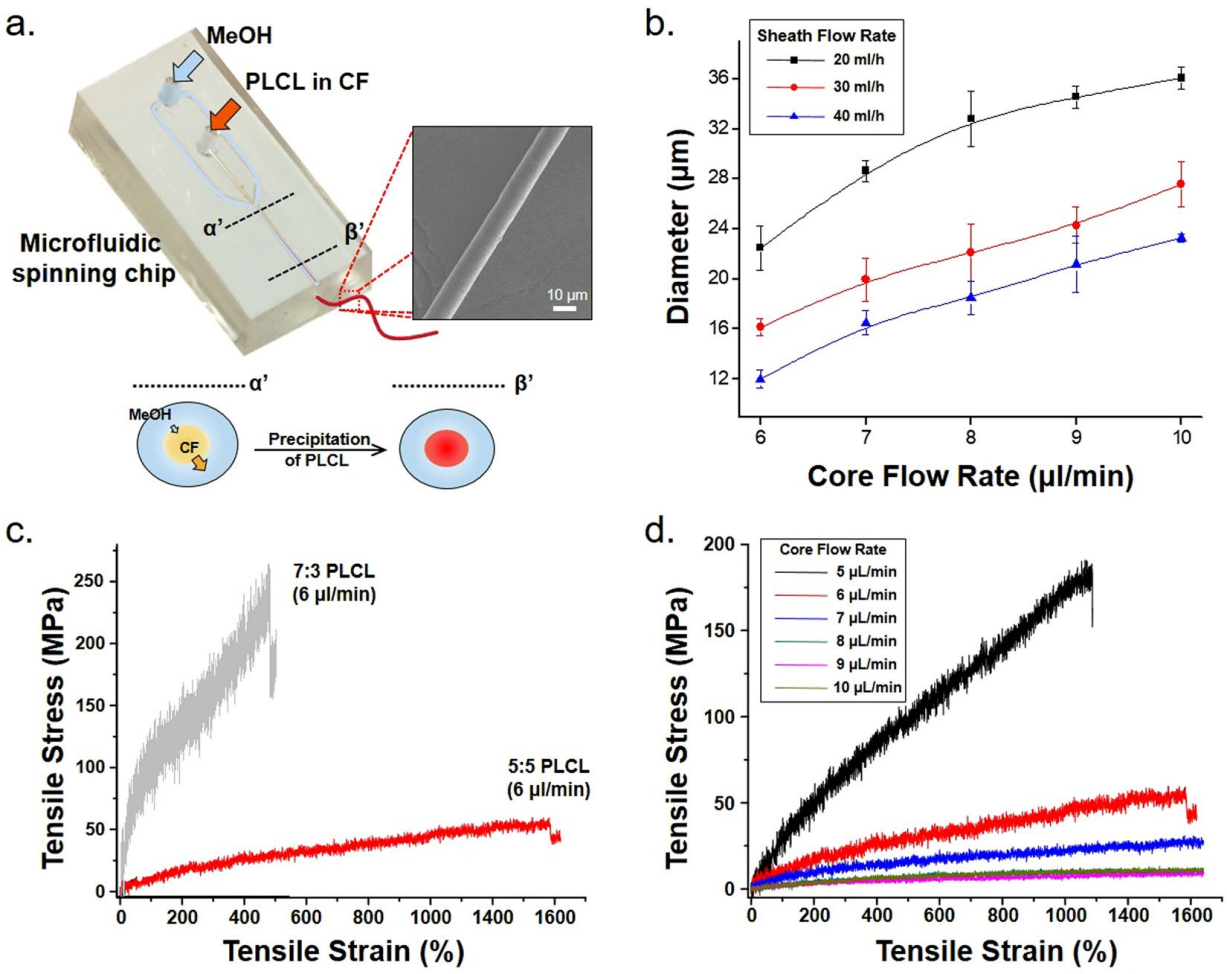
Fabrication and characterization of the mechanical properties of a single PLCL microfiber. (**a**) Fabrication of a single PLCL microfiber on a microfluidic chip. Introduction of PLCL in chloroform and methanol as core and sheath flows, respectively, caused the precipitation of PLCL within the outlet channel. (α′ → β′) (**b**) Diameters of single PLCL microfibers made using various core and sheath flow rates. (**c**) Strain-stress (SS) curves of single PLCL microfibers made with various relative amounts of PLCL, or (**d**) with various core flow rates with a fixed 20 mL/h sheath flow rate.

**Figure 3:**
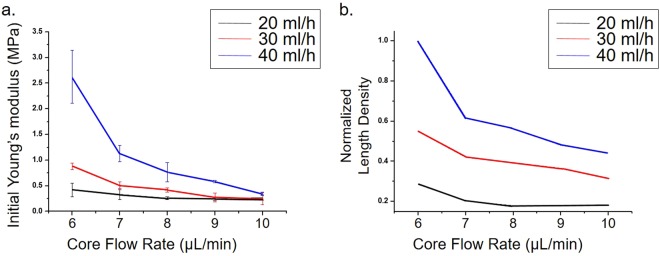
The relationship between iYm and length density. (**a**) Experimentally determined iYm values and (**b**) calculated length densities of microfibers made using core flow rates of 6, 7, 8, 9, and 10 μl/min with sheath flow rates of 20 mL/h, 30 mL/h, and 40 mL/h.

Feasible conditions were found that core flow rates from 5 μL/min to 10 μL/min were for the 5% (5:5)PLCL solution when sheath flow rates were 20 mL/h and 30 mL/h, while 6 μL/min to 10 μL/min for 5% (5:5) PLCL solution of sheath flow rates of 40 mL/h. The diameters of fibers fabricated under each feasible conditions were shown in Fig. 2b. Core flow rates from 6 μL/min to 8 μL/min were a feasible condition for the 5% (7:3)PLCL solution. The changes in an elongation at break, UTSH, UTSS, iYm of a single PLCL microfiber were shown in the SS curves. (Fig. 2c and d) The above mechanical properties associated with changing the molar ratio were derived from, respectively, the data in Fig. 2c and by comparing the data of Table 2 to the data of Table 3. Compared with 5% (5:5)PLCL microfiber, the 5% (7:3)PLCL microfiber showed overall higher UTSH, UTSS, and iYm values within the range of feasible conditions. On the other hand, the (5:5)PLCL microfiber showed a super-elastic increase of about 1200% for the first Instron loading length, but the (7:3)PLCL microfiber showed only an approximately 400% increase. Second, the changes in the SS curve and the above mechanical properties associated with changing the core flow rate (and keeping the sheath flow rate fixed at 20 mL/h) were derived from, respectively, the data in Fig. 2d and from the data of Table 2 and Table 3. Within the feasible conditions using the 5% (5:5)PLCL solution, the single microfiber diameter increased and the UTSH did not show any significant difference as the core flow rate was increased, while UTSS and iYm showed minimum values at 9 μL/min. Within the feasible conditions using the 5% (7:3)PLCL solution, as the core flow rate was increased, again the diameter increased and UTSH did not show a significant difference, while UTSS and iYm decreased. Also, when comparing the plot of experimentally determined iYm values (Fig. 3a) and length densities calculated according to suggested equation (Fig. 3b), similar trends were observed.

**Table 2:** Mechanical properties of single (5:5)PLCL microfibers made using various core flow rates with a fixed 20 mL/h sheath flow rate.

Core flow rate [μL/min]	Fiber Feasibility		Diameter [μm]	Ultimate Tensile Strength [N]	Ultimate Tensile Stress [MPa]	Initial Young’s Modulus [MPa]	Elongation at break [%]
	color^a^	Form^b^					
5	W^c^	Linear	21.05	0.0142	180.93	0.712	1100
6	W	Linear	22.50	0.0111	60.54	0.420	1346
7	W	Linear	28.66	0.0107	31.06	0.334	1284
8	W	Linear	32.81	0.0134	11.58	0.261	1307
9	W	Linear	34.56	0.0173	6.45	0.246	1257
10	W	Linear	36.08	0.0154	10.49	0.269	1203

^a^Color of the fibers when extruded from the outlet. ^b^Form of the microfiber extruded from the outlet of the microfluidic chip. ^c^Completely white.

**Table 3:** Mechanical properties of single (7:3)PLCL microfibers made using various core flow rates with a fixed 20 mL/h sheath flow rate

Core flow rate [μL/min]	Fiber Feasibility		Diameter [μm]	Ultimate Tensile Strength [N]	Ultimate Tensile Stress [MPa]	Initial Young’s Modulus [MPa]	Elongation at break [%]
	color	Form					
5	W	Not stable	—^a^	—	—	—	—
6	W	Linear	16.7	0.0541	246.8	2.107	416
7	W	Linear	20.6	0.0461	138	1.211	427
8	W	Linear	25.9	0.0509	96.6	1.097	430
9	V^b^	Linear	24.9	0.0364	74.7	1.101	369
10	T^c^	Linear	—	—	—	—	—

^a^ “−” indicates no measurement^b^, not convincing, and ^c^only a transparent stream of the core flow was observed.

**Figure 4:**
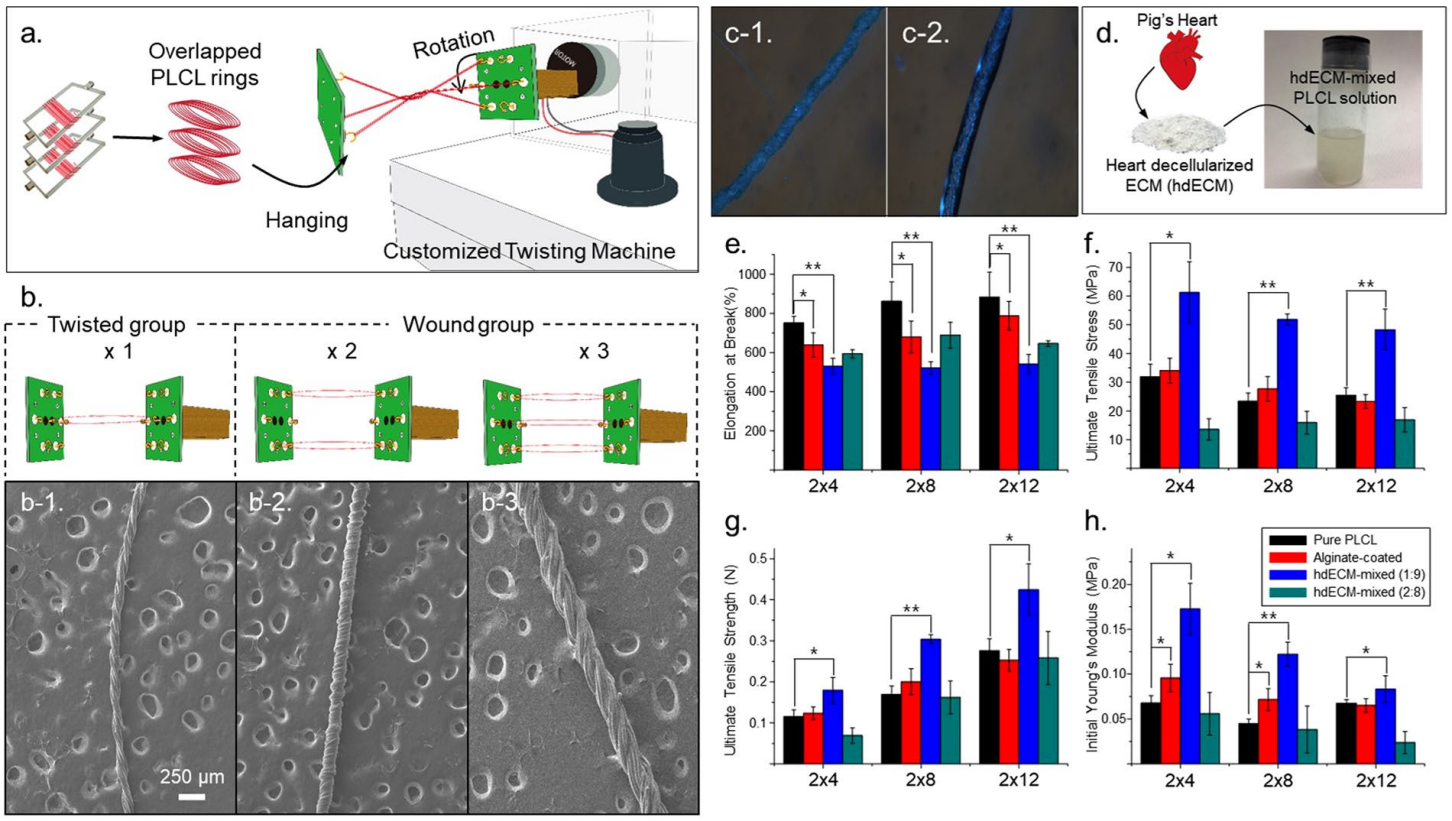
Fabrication and characterization of the mechanical properties of bundle PLCL fibers. (**a**) Schematic showing the fabrication of a bundle PLCL fiber using a CTM. b) Diagrams showing (**b**) one overlapped PLCL ring hung on the CTM in order to form a twisted group (left one column), or two or three rings on the CTM in order to form wound groups (right two columns). (**c**) Optical images of a bundle PLCL fiber (c-1) before and (c-2) after being coated with alginate. (**d**) Schematic of the method used to mix hdECM with a PLCL solution. (**e**–**h**) Mechanical properties, specifically (**e**) elongation at break, (**f**) UTSS, (**g**) UTSH, and (**h**) iYm, of bundle PLCL fibers made with various conditions (various numbers of microfibers, inclusion of a coating, and various ratios (1:9 and 2:8) of hdECM to PLCL. (n = 6 for each condition; *p < 0.05, **p < 0.001).

Bundle PLCL fibers of a twisted group and a wound group were well constructed using a CTM to see the effect of the number of single microfiber strands included in the bundle fiber. (Fig. 4a and b) Since pulling rates are important factors for mechanical response determination at this experiment, two conditions (10 mm/min and 5 mm/min) were applied for twisted groups. Furthermore, we also tested other ways to control the overall mechanical properties of the bundle fibers, including by coating them with alginate (Fig. 4c-1,c-2) or using microfibers formed after first mixing PLCL with porcine heart decellularized extracellular matrix (hdECM). (Fig. 4d) For the number of the single microfibers included in the bundle fiber, Table 4 shows the mechanical properties of the twisted and wound groups according to the number of single microfiber strands included in a bundle fiber. Most notably, the number of single microfiber strands included in the bundle fiber was found to have a negligible effect on the elongation at break, UTSS, and iYm. In contrast, UTSH was found to increase linearly with the number of single microfiber strands (R^2^ = 0.96). The effect of pulling rates on mechanical responses of bundle fiber was shown in Figure S4 that, first, the shape of SS curves differs that, before last shoot to the UTSS, they have quasi-plateau region whose values of stress are much smaller than those of 10 mm/min. Second, iYm values for group of 4 × 2 and 8 × 2 are bigger than those of 10 mm/min. As shown in Fig. 4e–h, the alginate-coated bundle fibers (red bars) showed slightly lower elongation at break and greater iYM values than did the pure bundle fibers (black bars), and no significant differences in UTSH and UTSS were measured between these fibers. In the case of the bundle fibers resulting from mixing hdECM with, the characterization was performed for fibers with a mass ratio of hdECM to (5:5)PLCL of 1:9 (blue bars in Fig. 4e–h) or 2:8 (green bars in Fig. 4e–h). The elongation at break for the 1:9 case was less than that for the pure bundle fiber; while the UTSH, UTSS, and iYM values for the 1:9 case were greater. However, the overall mechanical properties were worse for the fiber resulting from mixing hdECM and PLCL at a mass ratio of 2:8. This observation was attributed to the phase separation of the hdECM solution from the PLCL solution. Inhomogeneous mixing was observed, according to various experiments, in the mixed solution when more than 15% of the mass of the fiber was hdECM (data not shown). This observation implied an inhomogeneous distribution of PLCL molecules on a single microfiber, which can be explained as being due to a partial defect. Even worse mechanical properties were observed for a microfiber spun a week after mixing an hdECM solution and PLCL solution (Fig. S5).

**Table 4:** Mechanical properties of bundle PLCL fiber according to the number of single microfiber strands included within the bundle fiber.

Twisted group	4 × 2	8 × 2	12 × 2
Initial (~20%) Young’s Modulus [MPa]	0.06799/0.0081^a^	0.04250/0.00714	0.07/0.001
Ultimate Tensile Strength [N]^b^	0.1156/0.0160	0.169/0.0208	0.263/0.028
Ultimate Tensile Stress [MPa]	31.94/4.42	23.37/2.88	24.24/6.88
Elongation at break [%]	751/34	861/15	856/24
**Wound group (x2)**	**2 × 4 × 2**	**2 × 8 × 2**	**2 × 12 × 2**
Initial (~20%) Young’s Modulus [MPa]	0.077/0.006	0.0675/0.0044	0.06075/0.0051
Ultimate Tensile Strength [N]	0.130/0.0107	0.310/0.042	0.4478/0.02
Ultimate Tensile Stress [MPa]	18.01/5.78	21.39/6.37	20.548/2.02
Elongation at break [%]	839/39	703/42	751/45

^a^(average)/(standard deviation), ^b^newton.

**Figure 5:**
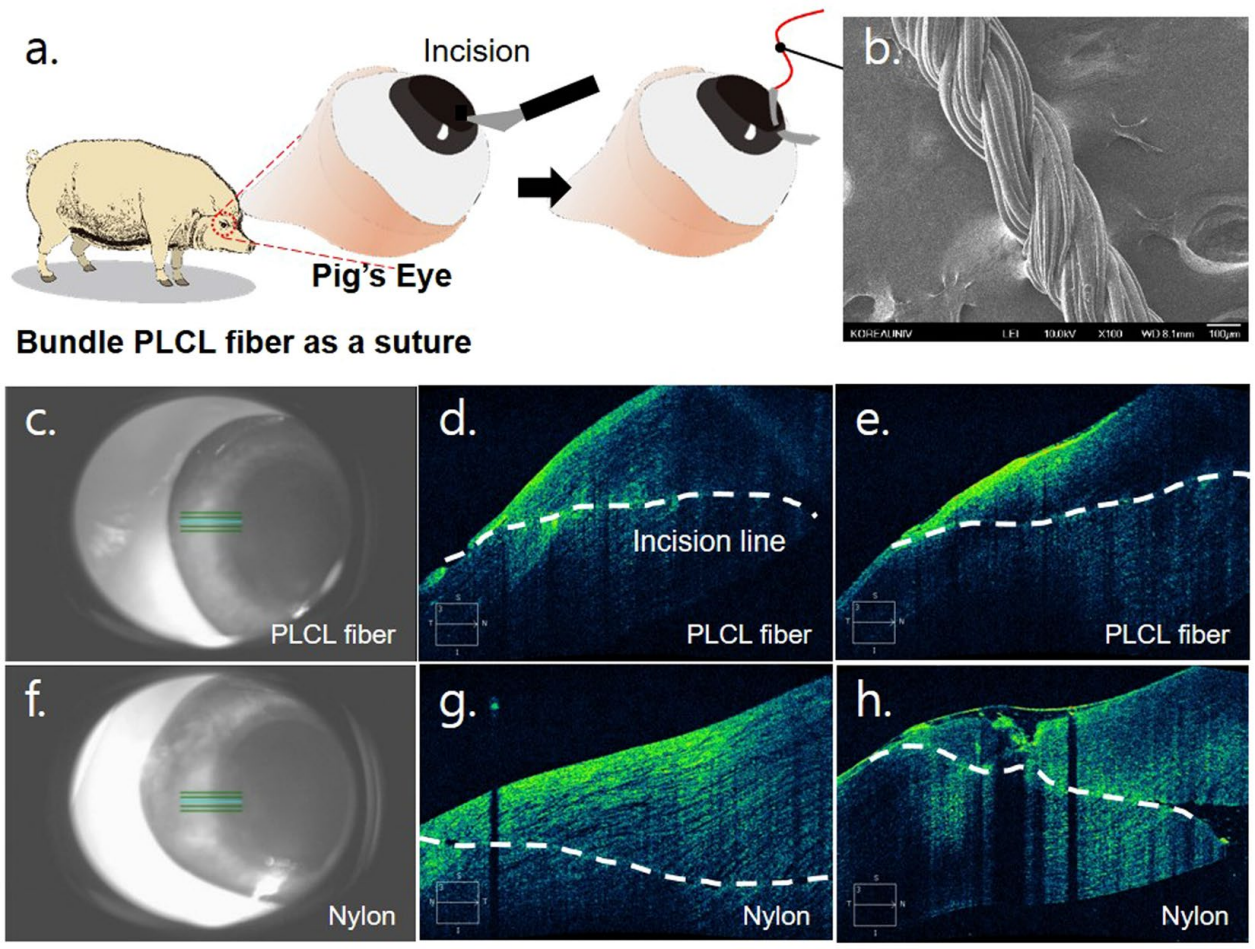
Use of bundle PLCL fiber as an ophthalmology suture (*ex vivo* test). (**a**) A schematic showing the *ex vivo* test procedure. (**b**) A SEM image of the bundle PLCL fiber used as the suture. We used a 2 × 8 bundle fiber that included hdECM in its microfibers and that showed an initial Young’s modulus of 0.125 MPa and UTSS of 50 MPa. (**c**–**h**) OCT images of the porcine eyes. Different parts (**c**–**e** and **f**–**h**) of the eye sutured with the bundle PLCL fiber (**d** and **g**) or nylon (**e** and **h**) were imaged.

An incision was made on the cornea (Fig. 5a) and was stitched up using the bundle PLCL suture (Fig. 5b) and a nylon 10–0 suture. No leakage was observed for both cases when injecting saline. One hour after *ex vivo* surgery, the incision line on the surface of the cornea was verified. (white dotted line in each of Fig. 5d,e,g and h) As shown in Fig. 5e and h, the cornea sutured with nylon10–0 showed a rough surface. On the other hand, when the bundle PLCL fiber was used as the suture, the post-operative cornea surface appeared smooth. We observed that the knots of the Nylon10–0 group and the PLCL group did not become untied for 5 days and ended the observation.

## Discussion

For a single PLCL microfiber, we have confirmed that not only the composition ratio but also the core flow rate change could vary the mechanical properties of microfluidic spinning microfiber. This difference shown between (5:5)PLCL microfibers and (7:3)PLCL microfiber may have been due to an increase in the amount of rigid L-lactic acid relative to caprolactone, but crystallization associated with the precipitation of (7:3)PLCL was apparently also an important factor. Also, we observed the plots of experimentally determined iYm and the length density in the fiber according to the equations we presented have consistent tendency. Since the equation (1) implies the length density of molecules along the axial direction of the microfiber to be determined by the ratio of the core flow rate to the sheath flow rate, and the area of the microfiber was previously indicated to be determined by the structure of the spinning chip and the flow rate^40^, we concluded that a microfluidic spinning chip can inherently control mechanical properties. Especially, our method in which a fiber is produced by precipitation within a chip has slow rate of fiber generation^41^, which gave time for a rearrangement of fiber molecules before perfect precipitation within the chip (note the definition of feasible condition), and was the main factor controlling the mechanical properties of the microfibers. The mechanical properties of a microfiber may thus be designed by simply using conventional spinning microchips as follows: changing the base materials to induce changes in certain mechanical properties such as elasticity, and changing the spinning flow condition to induce changes in the UTSS and Young’s modulus without significant changes in elasticity and UTSH. Thus, we concluded that when the conventional microfiber-spinning chip was used, we found the base material to be critical for determining the overall characteristics of the fibers; thus choosing the appropriate base material should be the first step in designing biomaterials that are intended to meet the need of the target tissues. Even with the same material, fine and independent control of the mechanical properties was shown to be possible using the microfluidic spinning chip.

For a bundle PLCL fiber, we were able to use a CTM to make a bundle fiber with different UTSH values, by changing the number of strands, while keeping the UTSS and iYm value relatively constant. Given that, of the twisted group, the angle of the internal microfiber with respect to the direction of the bundle fiber was found to have no significant relationship with measured mechanical properties, (Figure S7 and Supporting Information) the number of strands, rather than the angle of the microfiber within the bundle fiber, played a main role in controlling mechanical properties of the fiber in our system. The effect of pulling rate on the mechanical response of the fiber shows the properties of PLCL. Since PLCL is very elastic, it can be lengthened to about 800–900% of the initial length. In this regard, the different tendency of the SS curve seems to depend on the relation between the pulling rate, that is, the time that the force is given and the time required for the maximum deformation of the PLCL by the specific stress. Two remarkable characteristics at Figure S4, the quasi-plateau and different iYm for twisted groups of 4 × 2 and 8 × 2, may be due to similar or shorter time at which the force is applied to time at which the maximum deformation of the PLCL by the stress was done, unlike the case of 10 mm/min. We were also able to control mechanical properties of the fibers by coating them with biomolecules or blending biomolecules into them. Coating bundle PLCL fiber with alginate shows greater iYm values than did the pure bundle fibers with no other significant difference. This result may have been due to the difference in elongation at break of alginate and PLCL being so large that the values measured at the end of the elongation were not reflected in the alginate properties but the values for alginate were reflected in the iYM values. Also, less elongation at break, greater UTSH, UTSS, and iYm were shown for the 1:9 mixing case, while the overall mechanical properties were worse for the 2:8 mixing case. For 1:9 mixing case, These results resembled the differences between (5:5)PLCL and (7:3)PLCL. That is, mixing the hdECM with the PLCL solution changed the mechanical properties of the single microfiber itself, and this change caused a change in the mechanical properties of the bundle fiber. The change in the properties of the single microfiber might have been due to the filler effect, i.e., that hdECM acted as filler to improve the mechanical properties of the microfiber. Actually, many papers including our unpublished ones (in press) prove this^42,43^. While we originally might have expected hdECM to help harden the microfiber by either being a crystal nucleus or filler, we actually found that hdECM did not act as crystal nucleus and only acted as a filler to enhance the strength of the microfiber. As shown in Figure S6, significant changes were observed neither in the melting point nor in the glass transition temperature upon including hdECM, according to differential scanning calorimetry (DSC) results. For 2:8 mixing case, phase separation of the hdECM solution from the PLCL solution attributed to the results. Inhomogeneous mixing was observed, according to various experiments, in the mixed solution when more than 15% of the mass of the fiber was hdECM (data not shown). This observation implied an inhomogeneous distribution of PLCL molecules on a single microfiber, which can be explained as being due to a partial defect.

The observation of OCT images of the porcine cornea surface after surgery suggested that bundle PLCL fiber, applied as a suture, did not induce unmatched anastomotic strength and not induce the distortion of the surface structure while nylon suture could. This observation indicated that there was less scar formation, less of a delay in the wound healing process, or a lower concern that astigmatism developed when bundle PLCL fiber was used as a suture than when the nylon was used. These results implied that designing biomaterials to have mechanical strengths matching the mechanical strength of the target tissue is important when using biomaterials to maintain or restore tissue structure or function. Noting that the nylon10–0 surgical suture is one of the most commonly used sutures in ophthalmology surgery, the bundle PLCL fiber can be utilized as a suture and developed into a commodity. One hurdle of this method was that it was difficult to connect a bundle fiber to a fine surgical needle in the laboratory, and we had to inevitably attach the bundle fiber to a thick needle, which could increase needle-induced damage to tissue. However, this issue can be easily solved with a help of suture fabrication company in the near future.

## Conclusions

In summary, we developed a system which consists of a microfluidic spinning chip and a CTM, and where quite easily, we could independently control different mechanical properties of fibers. First, using a spinning chip, we were able to change the UTSS and iYm values while keeping the UTSH relatively constant. This ability was presumably due to the molecular arrangement of the base material in the fiber depending on the flow environment. Secondly, the relationship between the unit microfiber and bundle fiber in this bottom-up fabrication system can be summarized as follows: we were able to change the elongation at break, iYm, and UTSS of the bundle fiber by changing the properties of the unit microfiber itself, while we changed the UTSH of the bundle fiber mainly by changing the number of single microfibers in the bundle fiber. In other words, we have constructed bundle fibers whose mechanical properties can be tuned independently. We also conducted experiments in an applied case, and have shown once again the importance the of the mechanical design of the biomaterial for either case of being applied or being implanted. Overall, the current work has suggested an easy and novel method to control the mechanical properties of fiber-form biomaterials, which increased the range of potential applications of fibers by having developed a way to better control their mechanical properties.

## Electronic supplementary material

Supplementary Information
