# Author Correction: The use of microfluidic spinning fiber as an ophthalmology suture showing the good anastomotic strength control

**DOI:** 10.1038/s41598-018-31315-7

**Published:** 2018-08-31

**Authors:** DoYeun Park, In Sung Yong, Kyong Jin Cho, Jie Cheng, Youngmee Jung, Soo Hyun Kim, Sang-Hoon Lee

**Affiliations:** 1 KU-KIST Graduate School of Converging Science and Technology, Korea University, 145 Anam-ro, Seongbuk-gu, Seoul, 02841 Republic of Korea; 2 Department of Bio and Brain Engineering, KAIST, Daejeon, 34141 Republic of Korea; 3 Department of Ophthalmology, College of Medicine, Dankook University, 119 Danaeo-ro, Dongnam-gu, Cheonan-si, Chungcheongnam-do 31116 Republic of Korea; 4 MOE Key Laboratory of Spectrochemical Analysis & Instrumentation, Collaborative Innovation Center of Chemistry for Energy Materials, Key Laboratory for Chemical Biology of Fujian Province, State Key Laboratory of Physical Chemistry of Solid Surfaces, College of Chemistry and Chemical Engineering, Xiamen University, Xiamen, 361005 China; 5 Biomaterials Research Center, Korea Institute of Science and Technology, 5, Hwarang-ro 14-gil, Seongbuk-gu, Seoul, 02792 Republic of Korea; 6 School of Biomedical Engineering, College of Health Science, Korea University, 145, Anam-ro, Seongbuk-gu, Seoul, 02841 Republic of Korea

Correction to: *Scientific Reports* 10.1038/s41598-017-16462-7, published online 24 November 2017

The original version of this Article contained an error in the title of the paper, where the word “The” was incorrectly given as “Thae” and the word “microfluidic” was incorrectly given as “microfluic”.

Additionally, the original version of this Article contained an error in Affiliation 3, which was incorrectly given as ‘Department of Ophthalmology, College of Medicine, Dankook University, 119 Danaeo-ro, Dongnam-gu, Cheonan-si, Sungcheongnam-do, 31116, Republic of Korea’. The correct affiliation is listed below:

Department of Ophthalmology, College of Medicine, Dankook University, 119 Danaeo-ro, Dongnam-gu, Cheonan-si, Chungcheongnam-do, 31116, Republic of Korea

These errors have now been corrected in the PDF and HTML versions of the Article and in the accompanying Supplementary Information file.

