# Supplementary material for: The use of microfluidic spinning fiber as an ophthalmology suture showing the good anastomotic strength control
Source: Sci Rep. 2017 Nov 24;7:16264. doi: 10.1038/s41598-017-16462-7 (PMC5701120; doi:10.1038/s41598-017-16462-7)
Supplement: Supplementary file 1 — Supplementary Information [file 41598_2017_16462_MOESM1_ESM.docx]

**Type of article : Original Research**

**Corrected Title: The use of microfluidic spinning fiber as an ophthalmology suture showing the good anastomotic strength control**

*DoYeun Park, In Sung Yong, Kyong Jin Cho, Jie Cheng, Youngmee Jung, Soo Hyun Kim*, and Sang-Hoon Lee***

* : Corresponding Author

** : Deceased

**Supplementary data**


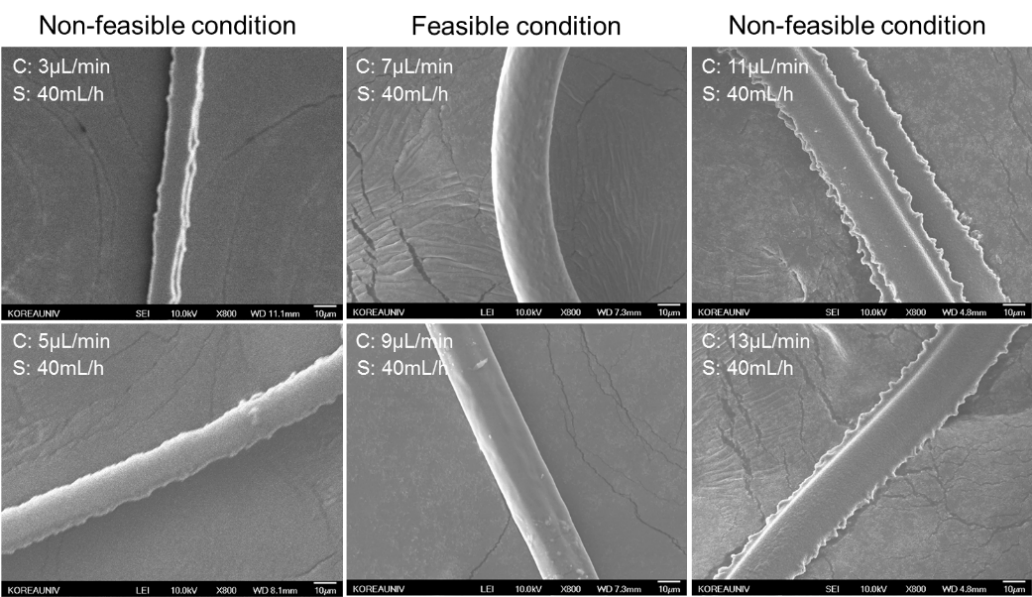


Figure S1. Scanning electronic microscope images of microfibers fabricated under feasible and non-feasible conditions. (See text for definition of “feasible condition”.)


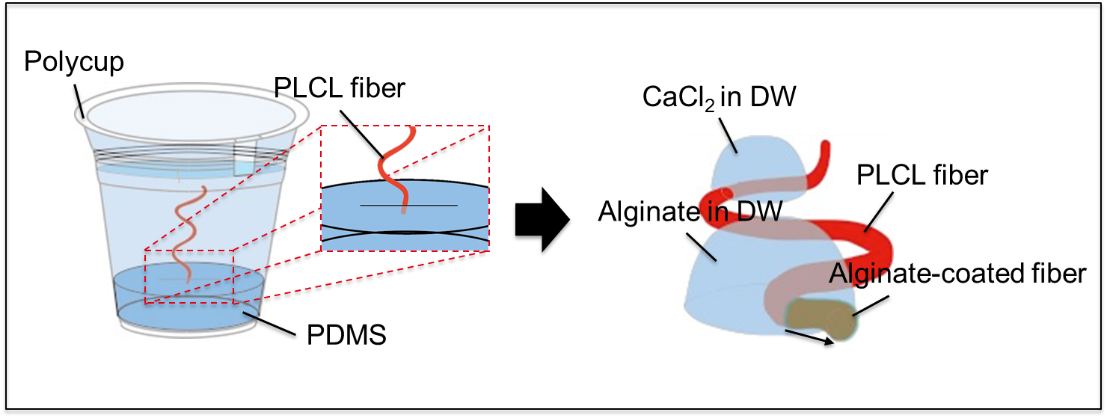


Figure S2. Alginate coating method


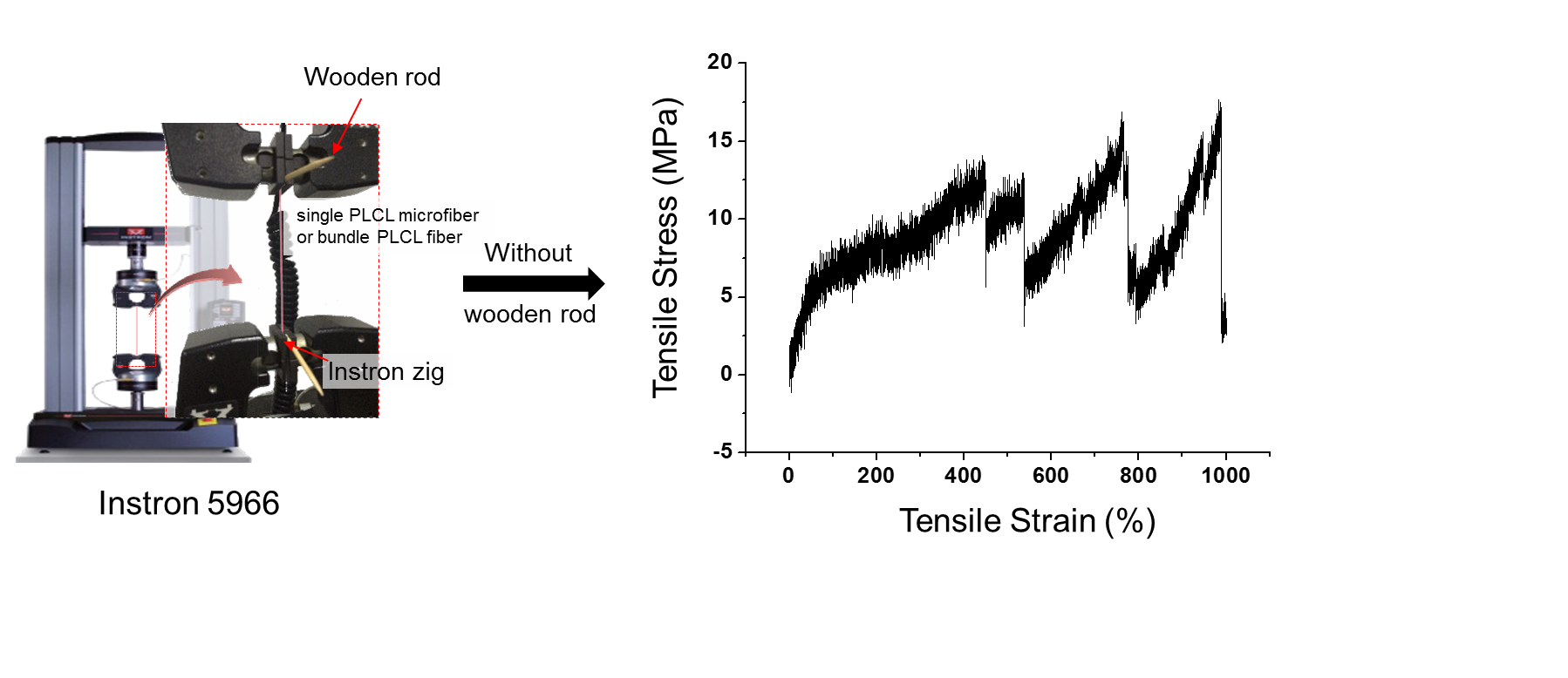


Figure S3. The distorted strain-stress curve obtained from measuring the mechanical properties of the microfiber without the wooden rod.


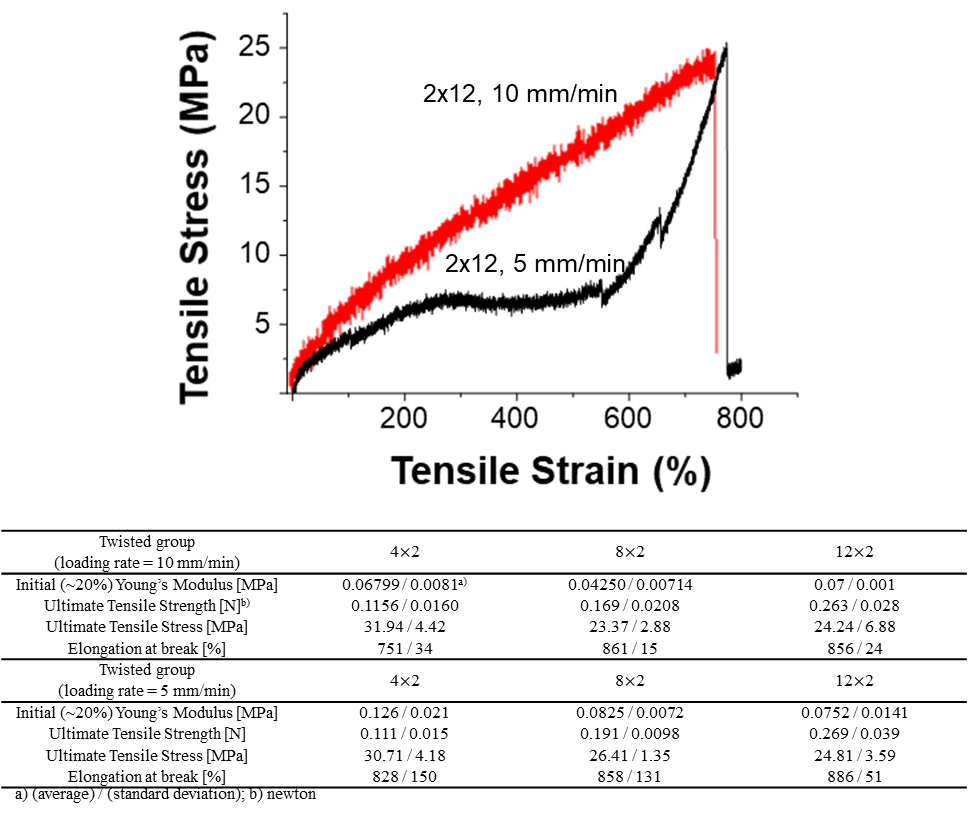


Figure S4. The effect of pulling rate on mechanical responses of bundle fiber.


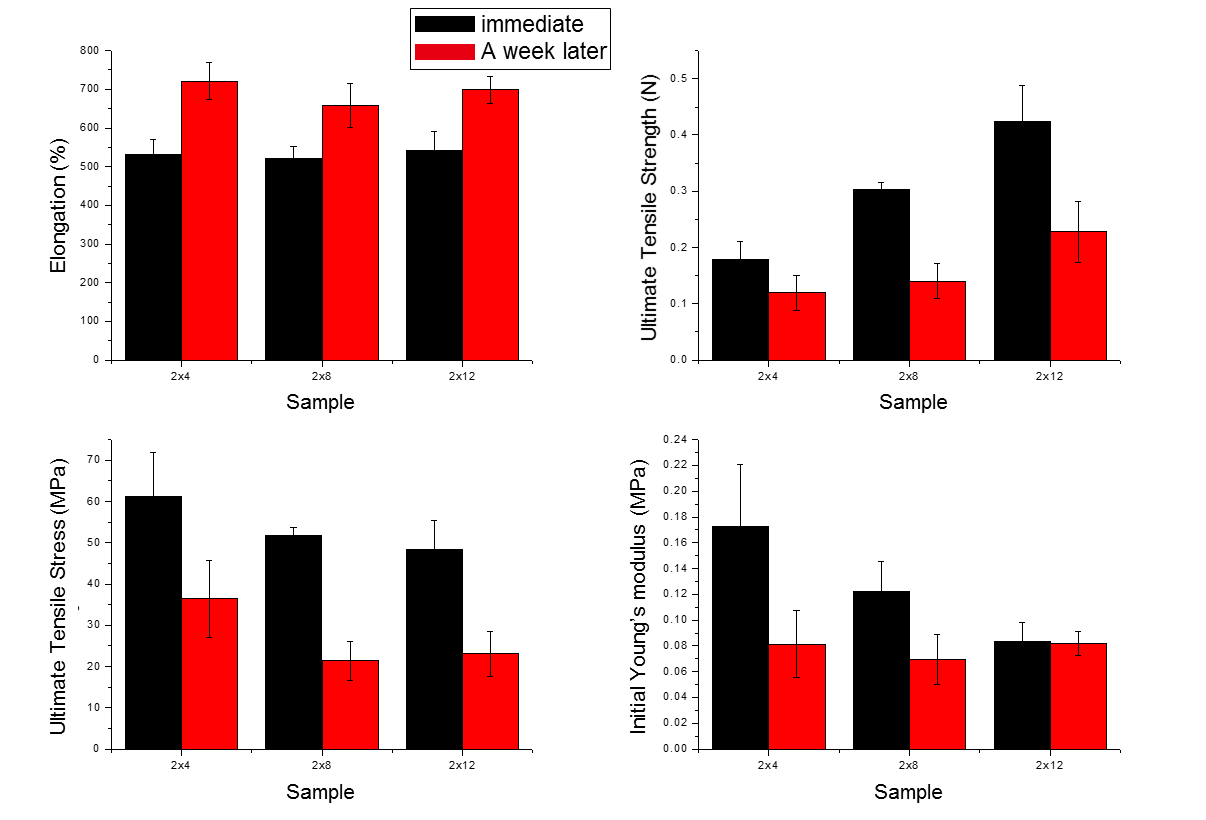


Figure S5. Mechanical properties of hdECM-mixed microfibers spun immediately or a week after mixing an hdECM solution and PLCL solution.


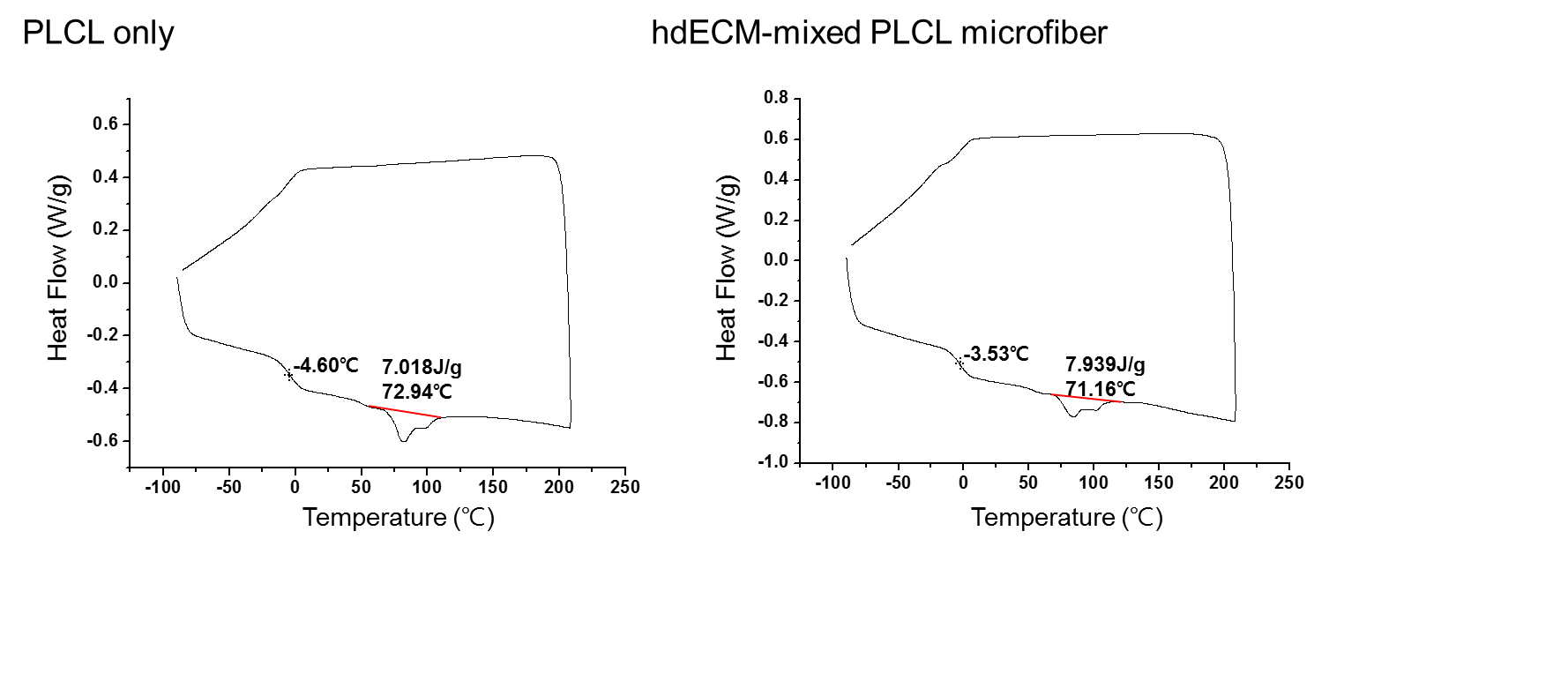


Figure S6. DSC thermograms to check the role of hdECM in improving the mechanical properties of the microfiber


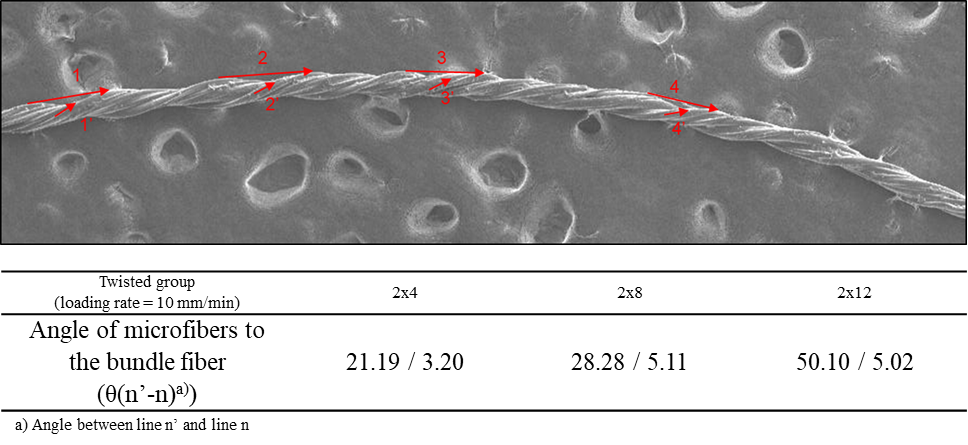


Figure S7. Measurement method for angles between internal microfibers and bundle fiber (SEM image, top) and the results (table, bottom)

Angle measurement and the correlation of angle with the measured mechanical properties

Observing the SEM images, the ridgeline where the bundle fibers twist occurs regularly. (Figure S7, top image) At this time, we could say that the angle between the internal microfiber and the bundle fiber is the angle between straight line (n) connecting the neighboring ridgeline and the straight tangent line (n’) to internal microfiber, drawn at the point where vertical line at the center of the line n meet the coaxial line of the bundle fiber. Repeat this at four locations to obtain the average value. (Figure S7, bottom image) Although the angle increase with the number of the microfibers within the bundle fiber, we concluded that this microstructural characteristic has small or no significant correlation with the measured mechanical properties given that UTSH had a linear correlation with the number of single microfibers within the bundle fiber (Table 4).
